# Effects of Appendectomy on the Onset and Course of Ulcerative Colitis in Chinese Patients

**DOI:** 10.1155/2018/2927891

**Published:** 2018-11-06

**Authors:** Dafan Chen, Jing Ma, Shengzheng Luo, Lungen Lu, Xinjian Wan, Qiwen Ben

**Affiliations:** ^1^Department of Gastroenterology, Shanghai General Hospital, Shanghai Jiao Tong University School of Medicine (originally named “Shanghai First People's Hospital”), Shanghai, China; ^2^Department of Gastroenterology, Ruijin Hospital, Shanghai Jiao Tong University School of Medicine, Shanghai, China

## Abstract

**Background:**

Previous epidemiological studies have suggested that appendectomy may be a protective factor against the development of ulcerative colitis (UC). However, the results of these studies were inconsistent, with rare studies in Chinese populations.

**Aim:**

This study examined the associations between appendectomy performed before UC diagnosis and the occurrence and clinical course of UC in Chinese patients.

**Methods:**

A case control study was conducted to compare the rate of appendectomy between UC patients and controls matched for age and sex at two Chinese hospitals. Clinical course of UC was compared between UC patients who underwent appendectomies before UC diagnosis and who did not.

**Results:**

402 UC patients and 402 controls were included. The percentage of appendectomy performed before UC diagnosis in UC patients did not differ significantly from controls (2.74% vs 3.98%, *P* = 0.442). Subgroup analysis on the basis of localization of UC patients did not find significant difference from controls. The extent of disease involvement in UC patients who underwent appendectomy was smaller than patients who did not (*P* = 0.009). Appendectomy was found to be significantly related to the location of the disease independent of smoking status in multivariate analysis (*P* < 0.001). Appendectomy did not influence severity of disease and need for immunosuppressive treatment or colectomy.

**Conclusion:**

We did not find a significant negative association between appendectomy and the UC occurrence in Chinese patients. Appendectomy performed before UC diagnosis may reduce the extent of UC involvement.

## 1. Introduction

Ulcerative colitis (UC) is one of the frequently encountered chronic inflammatory disorders of the colon and rectum. One important factor affecting the development of UC is the body's immune function [1, 2]. The appendix, as an immune organ, could alter the gut immune function and thereby influence the occurrence of UC. Some studies found that the opening of the appendix shows inflammation prior to the onset of UC [3]. Additionally, inflammation around the appendix is a frequent finding in UC patients [4–6]. Some epidemiological studies reported an inverse relationship between appendectomy and the incidence of UC [7–12], suggesting that appendectomy may be helpful in the prevention of UC. Several reports have suggested that appendectomy before UC diagnosis is associated with a less severe course of UC [8, 9, 13, 14]. However, this association could not be demonstrated consistently in the previous studies. Cohort studies from Denmark showed that the difference in the risk of developing UC between patients who had undergone appendectomy and controls was not significant [15]. In some studies, appendectomy performed before UC diagnosis did not reduce the severity of clinical course [10, 16, 17]. The clinical characteristics of UC vary among different ethnic groups [18–20]. Studies in China about the effect of appendectomy on UC development and especially its clinical course have been rarely reported. We performed this study to investigate the relationship between appendectomy performed before UC diagnosis and the occurrence and clinical course of UC in a Chinese population.

## 2. Patients and Methods

### 2.1. Study Design

This was a retrospective study conducted at two hospitals (Shanghai General Hospital and Ruijin Hospital) in Shanghai, China. The Investigation and Ethics Committee of Shanghai Jiao Tong University School of Medicine approved this study. A case-control study was undertaken to compare the incidence of appendectomy between UC patients and controls. UC patients who received treatment at these two hospitals between January 2015 and December 2017 were included. The relevant information was obtained from clinical interviews and medical charts. If detailed information regarding appendectomy and UC were not clear, the patients were contacted by questionnaire or telephone. UC was diagnosed based on the clinical, endoscopic, histological, and/or radiologic findings [21]. Appendectomy was considered as an operation of pure appendix. Dates of appendectomy and UC diagnosis were carefully assessed. The date of UC diagnosis was defined as the time of first detection of special abnormalities of the colon and rectum. According to each patient matching one control, age- (±2 years) and sex-matched individuals who visited the outpatient department of gastroenterology in these two hospitals during the same time period were included as controls. People with minimal gastrointestinal disease, such as dyspepsia, mild acute gastroenteritis, gastrointestinal polyp, peptic ulcer, and reflux esophagitis were included and inflammatory bowel disease (IBD) or suspected IBD were excluded from controls.

The age at UC diagnosis, extents of UC involvement, maximum disease activity, use of drugs, and colectomy were compared between appendectomy group in which patient underwent appendectomy prior to UC diagnosis and no appendectomy group in which patient did not. The results were used to analyze if appendectomy prior to UC diagnosis affected the clinical course of UC.

Demographic and clinical information were retrieved, including sex, date of birth, nationality (classified as Han nationality and minority), date of UC diagnosis, disease duration, history of appendectomy, smoking status (classified as nonsmoker if they never or rarely smoked and smoker), alcohol drinking (classified as nondrinker if they never or rarely drink and drinker), nonsteroidal anti-inflammatory drug (NSAID) use (classified as never used and used), maximum disease activity (assessed by Truelove-Witts criteria [22]), disease location (maximum extent recorded), and need for immunosuppressant (steroids, azathioprine, antitumor necrosis factor (TNF) antibodies, and so on) or surgical treatment history.

### 2.2. Statistical Analysis

Continuous data were presented as mean ± standard deviation and compared by Student's *t*-test. Chi square test or Fisher exact test was used to compare categorical variables. Paired data were compared in a pairwise-matched analysis. Trend test was used to compare intervals between UC diagnosis and appendectomy. Analysis of ordered categorical data was performed by the rank sum test. Smoking was controlled by regression analysis. A two-tailed *P* value <0.05 was considered to be statistically significant. All statistical analyses were conducted using SPSS, version 19.0 (SPSS, Chicago, IL).

## 3. Results

### 3.1. Clinical Characteristics

Overall, 417 UC patients visited these two hospitals during the study period. Due to the lack of specific information regarding appendectomy or UC, 15 UC patients were excluded. Finally, 402 UC patients were enrolled. Among these patients, 199 (49.50%) received immunosuppressive treatment and 28 (6.97%) had undergone colectomy. The percentages of cases of ulcerative proctitis, left-sided UC, and extensive UC were 9.95%, 42.29%, and 47.76%, respectively. Finally, 402 age- and sex-matched non-IBD individuals who visited the outpatient department of gastroenterology in these hospitals were enrolled as the controls. In controls who visited the department of gastroenterology, approximately 38% were dyspepsia patients, about 20% were peptic ulcer, and 17% were reflux esophagitis; the rest also included gastrointestinal polyp (15%) and mild acute gastroenteritis (10%). About one-third of the UC patients and controls were from outside Shanghai city.

### 3.2. Comparison of UC Patients with Controls

The incidence of appendectomy (2.74%) performed before UC diagnosis appeared lower than that in the controls (3.98%), but the difference was not significant (*P* = 0.442). No patient was undergone appendectomy after UC diagnosis. Subgroup analysis was performed on the basis of localization of UC patients. The analysis per stratum also did not find significant difference in appendectomy rate from controls (Table 1). Most of the patients (90.91%) were diagnosed >5 years after appendectomy (Table 2). The interval between appendectomy and UC diagnosis was 19.95 ± 10.29 years (range, 2.5–40 years) and was not significantly different to interval between appendectomy of a control and time of UC diagnosis of matched patient in trend test (*P* = 0.264). Appendectomy was performed at a mean age of 29.27 ± 9.35 years (range, 18–45 years) in the UC group, and only one patient underwent appendectomy before 20 years of age (Table 2). Previous study has indicated that only those patients who underwent appendectomy before 20 years gained protection to avoid occurrence of UC [23]. When appendectomy patients were classified into subgroups by age of appendectomy in 20 years, they were also not significantly different between UC group and controls (≤20 years, *P* = 0.123; >20 years, *P* = 0.807). The percentage of smokers in the UC group was significantly lower than that in the control group (*P* = 0.002). On multivariate analysis including smoking and appendectomy, smoking was found to be an independent protective factor for UC development in the logistic regression analysis (OR = 0.526; 95% CI = 0.381–0.725; *P* < 0.001), whereas appendectomy was not (OR = 0.741; 95% CI = 0.336–1.633; *P* = 0.457). A detailed comparison of the age, sex, nationality, smoking, alcohol drinking, and NSAID use between the UC patient and control groups is shown in Table 3.

### 3.3. Comparison of UC Patients with and without Appendectomy

The age at UC diagnosis in the appendectomy group was significantly higher than that in the no appendectomy group (48.64 ± 5.39 vs. 41.72 ± 15.70 years, *P* = 0.002). The extent of disease involvement was significantly greater in the no appendectomy group than in the appendectomy group among UC patients (*P* = 0.009). The incidence of proctitis was higher, and that of extensive colitis was lower in the appendectomy group compared with the no appendectomy group (Table 4). On the multivariate analysis including smoking and appendectomy using ordinal regression method, appendectomy was found to be a significant factor related to the extent of the disease independent of the smoking status (*P* < 0.001). There were no significant differences in sex, duration of disease, percentage of smoker, maximum disease activity, and the use of immunosuppressant drugs between appendectomy and no appendectomy groups. Although the difference in the colectomy rate between the appendectomy group and no appendectomy group was not significant, we found that no patient underwent colectomy in the appendectomy group. Detailed information regarding the clinical course of UC in the appendectomy group and no appendectomy group are provided in Table 4.

## 4. Discussion

Studies have suggested that the appendix is not a vestigial organ but rather an integral part of the gut immune system [24–26]. Its submucosal layer predominantly consists of B cells and CD4^+^ helper T cells, which may be the priming site for the immune response in colitis [27–29]. Appendectomy may influence the sampling of luminal antigen and T cell immunity. Epidemiological studies have shown an inverse relationship between appendectomy and UC development in some countries [7–12]. An animal study using T cell receptor *α* chain knockout mice showed that appendectomy could suppress colonic inflammation [27]. These findings suggest that appendectomy may be a protective factor for the development of UC. Meanwhile, genetic factors play a crucial role in the pathogenesis of UC. The incidence, risk factors, and clinical characteristics of UC vary greatly among different races [18–20]. Therefore, it is necessary to investigate the correlation between appendectomy and UC within different ethnic groups. Most of previous studies on the relationship between UC and appendectomy were performed on Caucasian populations, and the results of the relevant studies were inconsistent [10, 15–17]. The impact of appendectomy on the development and evolution of UC in Chinese population and most of other Asian populations is still unclear.

We undertook a retrospective case-control study when we analyzed the relationship between appendectomy performed before UC diagnosis and the occurrence of UC. The study showed that there was no significant correlation between appendectomy and the occurrence of UC, even in the subgroup analysis on the basis of localization of UC patients. The previous results from Chinese studies have been inconsistent. Jiang et al. found that appendectomy was a protective factor for development of UC [30]. However, Wang et al. did not find a significant association between appendectomy and the development of UC [31]. The prevalence of appendectomy in our UC group (2.74%) was not significantly different from that in the previously reported studies in China (1.69% [30] and 3.4% [31]) and was similar to the 2.57% prevalence in Korea [16] but significantly less than that reported in Japan and some Western countries [8–10]. The relationship between appendectomy and the development of UC was partially attributable to the prevalence of appendectomy in the control group. We chose clinic patients with minimal gastrointestinal disease as control, which was not considered to be related to appendectomy at present. This choice could reduce population selection bias compared to community control which was chosen in previous Chinese studies [30, 31]. The appendectomy rate in the control group (3.98%) of our study was different from that in the previous Chinese studies [30, 31] and was significantly lower than that in Japan and some Western countries. Multicenter studies with well design are needed to clarify the results in China. In addition, our study showed that most of the appendectomies were performed >5 years before UC diagnosis, which was similar to control. Thus, the effect of appendectomies is not related to interval between appendectomy and UC diagnosis, which was different to Crohn's disease (CD). Appendectomies were performed closely to date of CD diagnosis in CD [32]. Andersson et al. reported that protection from UC was found only in those patients who underwent appendectomy before the age of 20 years [23]. Although the trend of our results was in line with the data of Andersson et al., the difference did not reach significance which may be due to the small number of appendectomies in our study.

In this study, we also investigated the relationship between appendectomy and the clinical course of UC which was rarely studied in China. A large cohort study from North America revealed that appendectomy was not associated with a decreased severity of UC disease [17]. Previous studies from Korea and Australia showed that appendectomy did not influence UC clinical course [10, 16]. However, French researchers found that previous appendectomy was associated with a less severe course of UC [13]. A national cohort study from Europe showed that appendectomy before developing UC is associated with reduced colectomy risk and UC-related hospital admissions [14]. A prospective randomized multicenter trial to investigate the effect of appendectomy on the clinical course of UC has been carried out in Europe [33]. Our analysis indicated that the rate of proctitis was higher and extensive colitis was lower in the appendectomy group, and these results were independent of smoking status. This finding suggested that appendectomy before UC diagnosis may reduce the extent of UC involvement. This observation has not been reported in any of the previous Chinese studies. It is also worth mentioning that no patient underwent colectomy in the appendectomy group in our study, although the difference in the colectomy rate was not significant between the appendectomy group and no appendectomy group. Our study showed that the mean age at UC diagnosis was significantly higher in the appendectomy group than in the no appendectomy group. This may partly attribute to the fact that patients with appendectomy were older than those without appendectomy at the time of our study. Whether appendectomy may delay the onset of UC is still needed further investigation.

Andersson et al. found that appendectomy was protective against the risk of development of UC only in cases with appendicitis [23]. However, the study selected appendectomy as the sole criterion for inclusion and thus could not exclude a protective role for appendectomy. In animal models, appendectomy significantly suppressed the development of colitis even in animals that showed no evidence of appendicitis prior to surgery [27]. Cosnes et al. suggested that appendectomy, rather than inflammation of the appendix, may reduce the severity of UC in French where a significant proportion of patients had undergone appendectomy in the absence of appendicitis [13]. The above results suggested that appendectomy per se may be a protective factor for UC development independent of appendicular inflammation. The histopathology results for the removed appendixes were not all available in our study. Therefore, we did not perform subgroup analyses according to the cause of appendectomy.

Furthermore, our study investigated the smoking status in UC patients, which was considered as a strong environmental factor for UC [34–36]. We used regression analysis to control the effect of smoking while analyzing the effect of appendectomy on the UC occurrence and course. This study differentiated the patients and controls as nonsmokers and smokers. Due to lack of the specific information about smoking, we could not differentiate active smokers and exsmokers. Our results showed that the percentage of smokers in the UC group was significantly lower than that in the control group and being a smoker was a protective factor for UC development, which supported the findings of previous reports [34–36].

Our results should be interpreted in the context of the limitations of our study. The numbers of patients included in our study were not sufficiently large, and the number of appendectomies was small. Some data were not completely accessed, including the causes of appendectomy. This was a retrospective study, and only patients from tertiary care hospitals were included. Therefore, selection bias was difficult to avoid.

In conclusion, we analyzed the relationship between prior appendectomy and the occurrence and clinical course of UC, which was rarely studied in Chinese patients. We did not find a significantly negative association between appendectomy and UC occurrence. Our results showed that appendectomy before UC diagnosis may reduce the extent of UC involvement in Chinese patients.

## Figures and Tables

**Table 1:** Appendectomy status per localization in UC patients and controls.

Location of disease	All patients	Appendectomy (%)	Control (%)	*P* ^∗^
Proctitis	40	4 (10.00%)	2 (5.00%)	0.687
Left-sided colitis	170	5 (2.94%)	7 (4.12%)	0.774
Extensive colitis	192	2 (1.04%)	7 (3.65%)	0.180
Total	402	11 (2.74%)	16 (3.98%)	0.442

^∗^Pairwise matched analyses.

**Table 2:** The distribution of UC patients with varying age at appendectomy and interval between appendectomy and diagnosis of UC.

		Interval between appendectomy and UC diagnosis (years)		
		0-1	2–5	> 5
Age at appendectomy (years) in UC	≤20	0	0	1
	> 20	0	1	9

Age at appendectomy (years) in controls	≤20	0	1	4
	> 20	1	2	8

**Table 3:** Demographic characteristics.

	UC (*n* = 402)	Control (*n* = 402)	*P*
Age of UC diagnosis and control (years)	41.91 ± 15.55	42.21 ± 15.02	0.778
Male sex (%)	221 (54.98%)	221 (54.98%)	1.000
Han nationality (%)	386 (96.02%)	390 (97.01%)	0.442
Smokers (%)	85 (21.14%)	126 (31.34%)	0.002
Alcohol drinker (%)	64 (15.92%)	71 (17.66%)	0.509
NSAIDs use (%)	21 (5.22%)	16 (3.98%)	0.400

**Table 4:** Appendectomy vs no appendectomy in patients with UC.

	Appendectomy before UC diagnosis (*n* = 11)	No appendectomy (*n* = 391)	*P*
Age at UC diagnosis (years)	48.64 ± 5.39 (39–56)	41.72 ± 15.70 (16–81)	0.002
Median and range of disease duration (years)	5.66 ± 3.12	6.04 ± 5.08	0.807
Smokers (%)	2 (18.18%)	79 (20.20%)	0.869
Severity of disease			0.444
Mild (%)	5 (45.45%)	126 (32.23%)	
Moderate (%)	5 (45.45%)	231 (59.08%)	
Severe (%)	1 (9.09%)	34 (8.70%)	
Location of disease			0.009
Proctitis (%)	4 (36.36%)	36 (9.21%)	
Left-sided colitis (%)	5 (45.45%)	165 (42.20%)	
Extensive colitis (%)	2 (18.18%)	190 (48.59%)	
Immunosuppressant (%)	3 (27.27%)	192 (49.10%)	0.153
Colectomy	0	28 (7.16%)	1.000

## Data Availability

The data used to support the findings of this study are available from the corresponding author upon request.
